# New Medical College in Philadelphia

**Published:** 1847-02

**Authors:** 


*New Medical College in Philadelphia.*—A new Medical College in Philadelphia entitled the "Philadelphia College of Medicine," was chartered on the 14th ult, making the fifth Medical College in that ancient seat of Medical instruction. The faculty consists of four professors. Dr. Mitchell, of Transylvania University, Lexington, Ky., is to teach Theory and Practice, Midwifery and Medical Jurisprudence; James M'Clintock, M. D., Anatomy, Surgery, and Physiology. The first course is to be a Spring course, commencing in March next. We have no reluctance to tender our greetings to new additions to the already numerous Schools, provided, educational advantages, and the requisites for graduation are not lowered. We do not deem these results to follow necessarily from the fact that the number of schools is greater than the public wants require, as we will endeavor to show on some future occasion. We regret that the new Philadelphia College does not embrace a larger number of teachers, for we think it must be apparent that the *onus* of the branches which are combined, is too much for a single laborer. That Professors M'Clintock and Mitchell will do as much, and as well, as any others in discharging their aggregated duties we do not doubt, but human ability and strength have their limits, and we opine that before another session returns, the presence of other "Richmonds in the field" will be deemed desirable.

